# Divergent lineage of a novel hantavirus in the banana pipistrelle (*Neoromicia nanus*) in Côte d'Ivoire

**DOI:** 10.1186/1743-422X-9-34

**Published:** 2012-01-26

**Authors:** Laarni Sumibcay, Blaise Kadjo, Se Hun Gu, Hae Ji Kang, Burton K Lim, Joseph A Cook, Jin-Won Song, Richard Yanagihara

**Affiliations:** 1 Departments of Pediatrics and Tropical Medicine, Medical Microbiology and Pharmacology, John A. Burns School of Medicine, University of Hawaii at Manoa, Honolulu, HI 96813, USA; 2 Department of Biology, Université de Cocody, Abidjan 22, Côte d'Ivoire; 3 Department of Microbiology, College of Medicine, Institute for Viral Diseases, Korea University, Seoul 136-705, Republic of Korea; 4 Division of Enteric Bacterial Infections, Korea National Institute of Health, Cheongwon-gun, Chunngcheonngbuk-do 363-951, Republic of Korea; 5 Department of Natural History, Royal Ontario Museum, Toronto, ON M5S 2C6, Canada; 6 Department of Biology and Museum of Southwestern Biology, University of New Mexico, Albuquerque, NM 87131, USA; 7 Pacific Center for Emerging Infectious Diseases Research, John A. Burns School of Medicine, University of Hawaii at Manoa, 651 Ilalo Street, BSB320L, Honolulu, HI 96813, USA

**Keywords:** Hantavirus, Bat, Phylogeny, Côte d'Ivoire, Africa

## Abstract

Recently identified hantaviruses harbored by shrews and moles (order Soricomorpha) suggest that other mammals having shared ancestry may serve as reservoirs. To investigate this possibility, archival tissues from 213 insectivorous bats (order Chiroptera) were analyzed for hantavirus RNA by RT-PCR. Following numerous failed attempts, hantavirus RNA was detected in ethanol-fixed liver tissue from two banana pipistrelles (*Neoromicia nanus*), captured near Mouyassué village in Côte d'Ivoire, West Africa, in June 2011. Phylogenetic analysis of partial L-segment sequences using maximum-likelihood and Bayesian methods revealed that the newfound hantavirus, designated Mouyassué virus (MOUV), was highly divergent and basal to all other rodent- and soricomorph-borne hantaviruses, except for Nova virus in the European common mole (*Talpa europaea*). Full genome sequencing of MOUV and further surveys of other bat species for hantaviruses, now underway, will provide critical insights into the evolution and diversification of hantaviruses.

## Findings

Discovery of phylogenetically divergent hantaviruses in shrews and moles (order Soricomorpha, family Soricidae and Talpidae) [1-13] raises the possibility that rodents (order Rodentia, family Muridae and Cricetidae) may not be the principal or primordial reservoirs. Moreover, newfound hantaviruses harbored by soricomorphs of multiple species, distributed in widely separated geographic regions across four continents, suggest that their host diversity may be far more expansive than previously assumed. Specifically, other mammals having shared ancestry or ecosystems with soricomorphs may serve as reservoirs and may be important in the evolutionary history and diversification of hantaviruses. In particular, bats (order Chiroptera) may be potential reservoirs by virtue of their rich diversity and vast geographical range, as well as their demonstrated ability to host myriad medically important, disease-causing viruses [14-18]. Surprisingly little attention, however, has been paid to this possibility.

As in our previous investigations on the spatial and temporal distribution of hantaviruses in soricomorphs [2-13], we relied on the availability of archival tissues. Using the PureLink Micro-to-Midi total RNA purification kit (Invitrogen, San Diego, CA), total RNA was extracted from 168 frozen and 45 ethanol-fixed liver and other visceral tissues of 213 insectivorous bats (representing 13 genera), collected during May 1981 to June 2011 in Asia, Africa and the Americas (Table 1). cDNA was then prepared with the SuperScript III First-Strand Synthesis System (Invitrogen) using random hexamers, and PCR was performed as described previously, using an extensive panel of oligonucleotide primers, designed on conserved genomic sequences of rodent- and soricomorph-borne hantaviruses [2-13,19,20]. Each reaction mixture contained 250 μ dNTP, 2 mM MgCl_2_, 1 U AmpliTaq polymerase (Roche, Basel, Switzerland) and 0.25 μ oligonucleotide primers. Initial denaturation at 94°C for 5 min was followed by two cycles each of denaturation at 94°C for 40 s, two-degree step-down annealing from 48°C to 38°C for 40 s, and elongation at 72°C for 1 min or 1 min 20 s, then 32 cycles of denaturation at 94°C for 40 s, annealing at 42°C for 40 s, and elongation at 72°C for 1 min, in a GeneAmp PCR 9700 thermal cycler (Perkin-Elmer, Waltham, MA). Amplicons were purified using the QIAQuick Gel Extraction Kit (Qiagen, Hilden, Germany), and DNA sequencing was performed using an ABI Prism 377XL Genetic Analyzer (Applied Biosystems, Foster City, CA).

**Figure 1:**
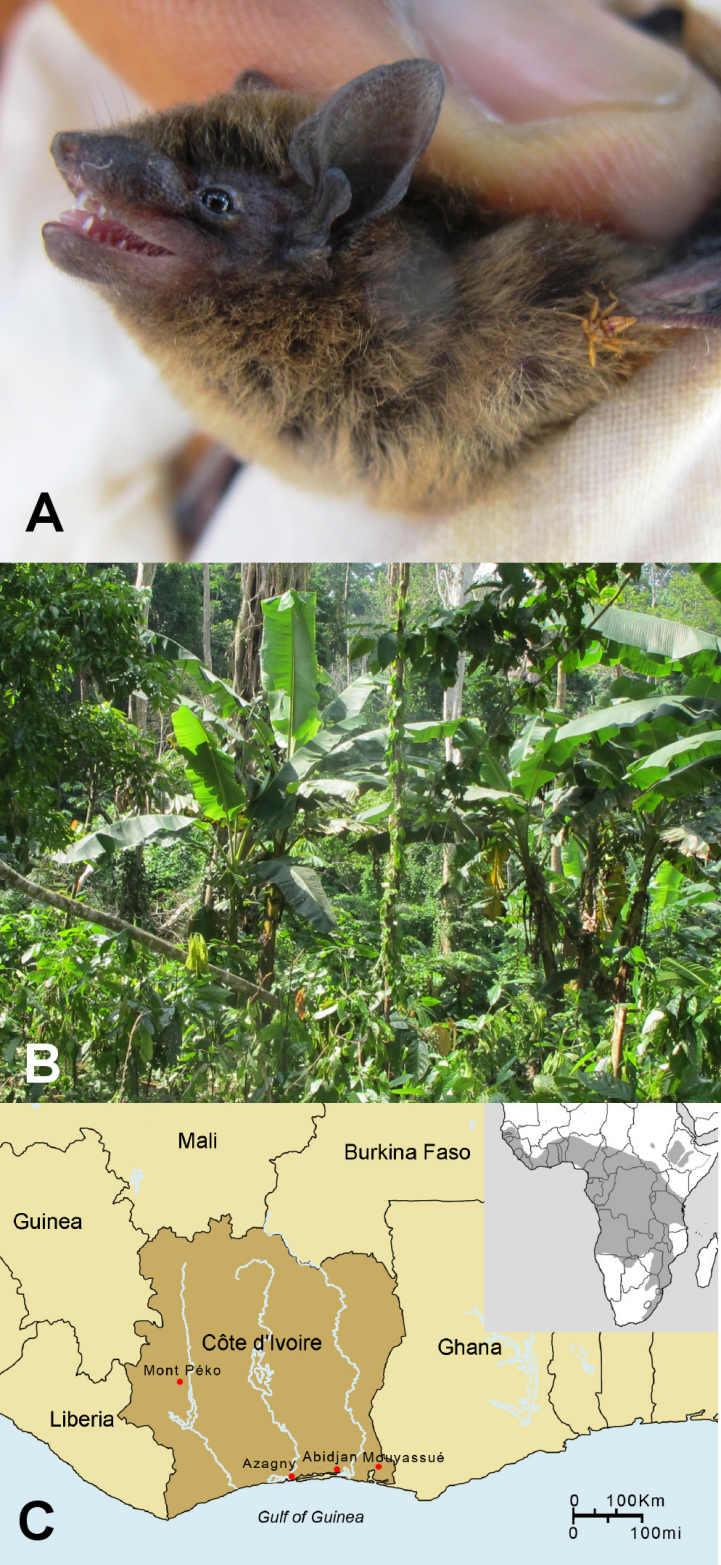
**(A) Banana pipistrelle (*Neoromicia nanus*) in which hantavirus RNA was detected**. (B) Capture site of banana pipistrelles near Mouyassué village in Aboisso District. (C) Map of Côte d'Ivoire, showing Mouyassué, Azagny and Mont Péko, where insectivorous bats were captured. The geographic range of the banana pipistrelle extends throughout sub-Saharan Africa (shaded area in inset).

After innumerable failed attempts, hantavirus RNA was detected by RT-PCR in ethanol-fixed liver tissues from two of 12 banana pipistrelles (*Neoromicia nanus *Peters 1852), captured during June 2011 near Mouyassué village (5°22'07"N, 3°05'37"W) in Aboisso District, 130 km from Abidjan, in the extreme southeastern region of Côte d'Ivoire in West Africa (Figure 1). The taxonomic identity of the hantavirus-infected vesper bats was confirmed by phylogenetic analysis of the cytochrome *b *gene of mtDNA (GenBank JQ287717), amplified by PCR as previously described [8,9]. Despite similarly exhaustive efforts, hantavirus RNA was not detected in any of the other bat species tested (Table 1), including frozen liver tissue of six tiny pipistrelles (*Pipistrellus nanulus*), collected in Parc National du Mont Péko, 700 km northwest of Mouyassué, in February 1992, and ethanol-fixed liver tissue of three tiny pipistrelles, collected in December 2009 in Azagny, where a hantavirus was previously found in the West African pygmy shrew (*Crocidura obscurior*) [8].

**Table 1:** Detection of hantavirus RNA in tissues of insectivorous bats by RT-PCR

Genus species	USA	Bolivia	Guyana	Liberia	Côte d'Ivoire	Mongolia	Malaysia	Total
*Antrozous pallidus*	0/20							0/20
*Corynorhinus townsendii*	0/19	0/1						0/20
*Eptesicus fuscus*	0/21							0/21
*Eptesicus gobiensis*						0/20		0/20
*Eptesicus sp*.				0/4				0/4
*Hipposideros cafer*				0/14	0/5			0/19
*Hipposideros cervinus*							0/11	0/11
*Hipposideros cyclops*					0/11			0/11
*Hipposideros gambianus*				0/5				0/5
*Lasiurus cinereus*	0/20							0/20
*Mops condylurus*					0/2			0/2
*Neoromicia nanus*					2/12			2/12
*Nycteris arge*					0/1			0/1
*Nycteris major*					0/1			0/1
*Nycteris thebaica*					0/1			0/1
*Pipistrellus nanulus*					0/9			0/9
*Pteronotus parnellii*			0/5					0/5
*Rhinolophus trifolatus*							0/8	0/8
*Scotophilus sp*.					0/3			0/3
*Tadarida brasiliensis*	0/10	0/10						0/20
Total	0/90	0/11	0/5	0/23	2/45	0/20	0/19	2/213

A 423-nucleotide region of the RNA-dependent RNA polymerase-encoding L segment, amplified using a hemi-nested primer set (outer: 5'-GAAAGGGCATTNMGATGGGCNTCA GG-3', 5'-AACCADTCWGTYCCRTCATC-3'; inner: 5'-GNAAAYTNATGTATGTNAGT GC-3', 5'-AACCADTCWGTYCCRTCATC-3'), was aligned and compared with hantavirus sequences available in GenBank, using ClustalW (DNASTAR, Inc., Madison, WI) [21] and transAlign [22]. The newfound hantavirus, designated Mouyassué virus (MOUV), exhibited low nucleotide and amino acid sequence similarity of less than 69% to all representative soricomorph- and rodent-associated hantaviruses, except for the 76.3% sequence similarity with Nova virus (NVAV), previously reported in the European common mole (*Talpa europaea*) [12]. Interestingly, MOUV sequences were identical in the two banana pipistrelles (KB576 and KB577), a male-female pair captured simultaneously and presumed to be a mating couple, suggesting horizontal virus transmission or common-source infection.

**Figure 2:**
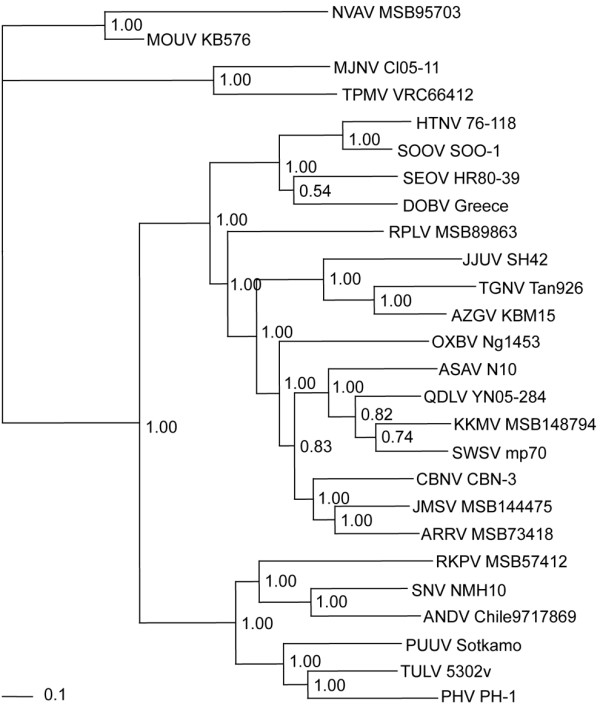
**Phylogenetic trees were generated by maximum-likelihood and Bayesian methods, under the GTR+I+Γ model of evolution, based on a 423-nucleotide L-genomic segment of Mouyassué virus (MOUV KB576) (GenBank **JQ287716). Since tree topologies were similar using RAxML, PAUP* and MrBayes, the tree generated by MrBayes was displayed. The numbers at each node are posterior probabilities. The scale bar indicates nucleotide substitutions per site. The phylogenetic position of MOUV is shown in relation to representative soricomorph-borne hantaviruses, including Thottapalayam virus (TPMV VRC66412: EU001330) from the Asian house shrew (*Suncus murinus*), Imjin virus (MJNV Cl05-11: EF641806) from the Ussuri white-toothed shrew (*Crocidura lasiura*), Jeju virus (JJUV SH42: HQ663935) from the Asian lesser white-toothed shrew (*Crocidura shantungensis*), Tanganya virus (TGNV Tan826: EF050454) from the Therese's shrew (*Crocidura theresae*), Azagny virus (AZGV KBM15: JF276228) from the West African pygmy shrew (*Crocidura obscurior*), Cao Bang virus (CBNV CBN-3: EF543525) from the Chinese mole shrew (*Anourosorex squamipes*), Ash River virus (ARRV MSB73418: EF619961) from the masked shrew (*Sorex cinereus*), Jemez Springs virus (JMSV MSB144475: FJ593501) from the dusky shrew (*Sorex monticolus*), Seewis virus (SWSV mp70: EF636026) from the Eurasian common shrew (*Sorex araneus*), Kenkeme virus (KKMV MSB148794: GQ306150) from the flat-skulled shrew (*Sorex roboratus*), Qiandao Lake virus (QDLV YN05-284: GU566021) from the stripe-backed shrew (*Sorex cylindricauda*), Camp Ripley virus (RPLV MSB89863: EF540771) from the northern short-tailed shrew (*Blarina brevicauda*), Asama virus (ASAV N10: EU929078) from the Japanese shrew mole (*Urotrichus talpoides*), Oxbow virus (OXBV Ng1453: FJ593497) from the American shrew mole (*Neurotrichus gibbsii*), Rockport virus (RKPV MSB57412: HM015221) from the eastern mole (*Scalopus aquaticus*), and Nova virus (NVAV MSB95703: FJ593498) from the European common mole (*Talpa europaea*). Also shown are rodent-borne hantaviruses, including Hantaan virus (HTNV 76-118: NC_005222), Soochong virus (SOOV SOO-1: DQ562292), Dobrava virus (DOBV Greece: NC_005235), Seoul virus (SEOV HR80-39: NC_005238), Tula virus (TULV M5302v: NC_005226), Puumala virus (PUUV Sotkamo: NC_005225), Prospect Hill virus (PHV PH-1: EF646763), Andes virus (ANDV Chile-9717869: NC_003468), and Sin Nombre virus (SNV NMH10: NC_005217).

MOUV formed a uniquely divergent lineage, distant from all other hantaviruses identified to date, except for NVAV (Figure 2), in phylogenetic trees based on L-segment sequences, generated by the maximum-likelihood and Bayesian methods, implemented in PAUP* (Phylogenetic Analysis Using Parsimony, 4.0b10) [23], RAxML Blackbox webserver [24] and MrBayes 3.1 [25], under the best-fit GTR+I+Γ model of evolution established using jModeltest 0.1.1 [26]. Topologies were well supported by bootstrap analysis of 100 iterations, and posterior node probabilities based on two runs each of 2 million generations sampled every 100 generations with burn-in of 25%.

Despite the overall success of our brute-force RT-PCR approach at identifying previously unrecognized hantaviruses in frozen tissues [2,3,5-7,10-13] and tissues preserved in RNAlater^® ^RNA Stabilization Reagent [4,8], designing universal primers for the amplification of soricomorph-borne hantaviruses has presented continuing challenges. Thus, while it is likely that many more hantaviruses await discovery, overcoming technical barriers is essential to facilitating their detection. Viewed in this context, the failure to detect hantavirus RNA in all but one bat species was not altogether unexpected and may be attributed simply to suboptimal primer design and imperfect cycling conditions. Also, low RNA yields and poor RNA preservation in tissues fixed in ethanol under field conditions may have thwarted our efforts at obtaining more of the MOUV genome. That said, the successful amplification of hantavirus RNA from ethanol-fixed tissues is highly instructive and augments the pool of archival tissues for future exploratory studies of hantaviruses in bats, and possibly other insectivorous small mammals that share ancestral lineages with soricomorphs, such as hedgehogs (order Erinaceomorpha, family Erinaceidae).

Dating to the seminal discovery of Hantaan virus in lung tissue of the striped field mouse (*Apodemus agrarius*) [27], lung has been the preferred tissue in studies aimed at finding new hantaviruses [28-30]. However, lung is not the only tissue in which hantaviruses can be detected [27,31]. In our search of genetically distinct hantaviruses in long-stored archival tissues from shrews and moles, lung tissue was frequently unavailable. Instead, liver tissue was more often accessible and proved to be quite suitable [4,5,12,13]. Similarly, liver tissues were more often available in the present study. As in reservoir rodents and soricomorphs, hantavirus RNA is likely to be present in many tissues of persistently infected bats. Real-time quantitative RT-PCR analysis of lung, liver and other viscera will clarify the tissue distribution of MOUV in newly captured banana pipistrelles from Mouyassué.

Having their fossil origins in the Eocene epoch, approximately 50 million years before present, bats occur on every continent except Antarctica and are among the most speciose orders of mammals, with more than 1,100 extant species [32]. The banana pipistrelle, which is distributed widely in forests and savannas across sub-Saharan Africa (Figure 1C, inset), is one of 13 species in the genus *Neoromicia *of the family Vespertilionidae and subfamily Vespertilioninae. Like other vesper bats, the banana pipistrelle is insectivorous. Unlike large fruit bats, such as the straw-colored fruit bat (*Eidolon helvum*) and hammer-headed bat (*Hypsignathus monstrosus*), which are sold as bush meat, the banana pipistrelle, weighing approximately 3 g, is not consumed as food. However, because banana pipistrelles occasionally roost within houses or reside near human habitation, rare human encounters raise the possibility of hantavirus exposure.

Previously, serological evidence of hantavirus infection was reported in the common serotine (*Eptesicus serotinus*) and greater horseshoe bat (*Rhinolophus ferrumequinum*) captured in Korea [33], but genetic analysis of hantaviral isolates from these insectivorous bat species proved to be indistinguishable from prototype Hantaan virus [34], suggesting laboratory contamination. In the present study, the strikingly divergent lineage of MOUV precluded any possibility of contamination and lends support to our earlier conjecture that the ancient origins of hantaviruses may have involved insect-borne viruses [7,10], with subsequent adaptation to and host switching between early soricomorph and chiropteran ancestral hosts in the mammalian superorder Laurasiatheria. However, since the biological and evolutionary implications of bats as reservoirs of hantaviruses are considerable, studies are underway to establish that the banana pipistrelle is the natural host of MOUV. Moreover, high-throughput sequencing technology is being applied to obtain the full genome of MOUV and to ascertain the geographic range and genetic diversity of hantaviruses harbored by bats.

## Competing interests

The authors declare that they have no competing interests.

## Authors' contributions

LS and SHG independently performed RNA extraction, RT-PCR and DNA sequencing reactions. HJK and JWS provided primer design and phylogenetic analysis. BK, BKL and JAC provided tissues and information about bats. RY conceived the project and provided overall scientific oversight. All authors contributed to the preparation of the final manuscript.
